# Creasote Inhalation, in Phthisis

**Published:** 1853-10

**Authors:** Thomas Inman


### *Creasote Inhalation in Phthisis, and Bronchitis.*

BY THOMAS INMAN, M. D.

Sir: Will you permit me to call the attention of the profession to a plan of treatment which I have found of great benefit in many diseases of the chest. I believe that it has been tried before, but I cannot at present lay my hand upon any account respecting it. I allude to the inhalation of the vapor of creasote combined with steam.

The first case in which I employed it was one of phthisis, in which the cough was peculiarly distressing, in consequence of the presence of necrosis and suppuration in both ears, which were attended with severe and constant throbbing pain. A host of opiates were tried, one after another, but had to be given up, from the sickness and headache they produced. I tried creasote inhalation on speculation; and it answered so remarkably well, that its use was continued to within a few days of death. The patient always expressed himself as very grateful for the relief it gave to the cough.

Encouraged by the success attending this, I determined to try it in other cases, some of chronic bronchitis, the majority of phthisis. In all I have found it most useful in allaying cough, and checking secretion and expectoration. By allaying the cough, and thus increasing the comfort of the patient, it seems in some degree to increase the strength; at any rate, it saves its diminution. It does not of itself appear to possess any curative power. As a palliative, it is useful in all cases; and I have heard it spoken of most gratefully by a patient who only lived to use it for three or four days.

The mode of using it is very simple. I direct from four to ten drops of creasote to be placed in an old teapot, and a small quantity of boiling water to be poured over it. The spout is to be protected by a piece of flannel, and the steam inhaled through that until it begins to feel cool. Care must be taken, of course, not to put too much water in. This answers well enough in poor practice. In hospital and private practice more elegant apparatus may be used. An inhaler made by Weiss answers remarkably well. It consists of a closed tin can, holding about three pints; at the top are two large tubes which communicate with the interior; one is furnished with an ivory mouth-piece, to prevent the lips being burned by the hot metal; the other is turned upwards, and through this the creasote and water are readily introduced. The size of the tubes makes the inhalation of the steam peculiarly easy. When this is employed I find that the temperature of the water should not exceed 150 degrees. The immediate effects are a feeling of warmth about the throat, and a sensation "as if you had lungs under your ribs," followed by reduced irritability of the mucous membrane, and subsequently by evidence of the creasote having entered the blood.

It would take up too much space were I to detail all the cases in which I have employed it in hospital and private practice; one will suffice, and will serve to indicate the routine treatment I have adopted in phthisis.

A. B., seaman, aged 30, was admitted into the Northern Hospital with severe cough, dyspnœa, and profuse expectoration. He had been ill for three months, during which he had emaciated considerably. The pulse was 130, and there were copious night sweats. On examining the chest, I found solidification of the apex of the left lung, and coarse mucous rales all over the same side, both anteriorly and posteriorly. At the apex of the right lung there was tubular breathing in one spot, and prolonged expiration in another. The expectoration was of thick, semi-transparent mucus, free from air bubbles, and mixed here and there with grey tubercular patches. He was ordered to have creasote inhalation three or four times, and to take internally half an ounce of cod-liver oil, with twenty-five minims of tincture of iron, three times daily. The severity of the symptoms abated in the week, and in six weeks the man was convalescent. The right lung now seems perfectly healthy; all traces of solidification have disappeared from the apex of the left; the respiration there, and over the whole of that lung, has become natural. The pulse has come steadily down to 72. The man has gained flesh, and the capacity of the chest, as evidenced by the power to take in a deep inspiration, has signally increased.

I may mention, that the use of iron has been adopted in consequence of the effect it had on a little girl, who had been previously employing creasote inhalation and cod-liver oil for some weeks without any indication of improvement. In her case, the whole of the left, and the upper part of the right lung were implicated: but I could not detect any cavities. The case seemed hopeless, when she began with the tincture of the sesquichloride, in addition to her other medicine. In two days an improvement was seen; in four it was confirmed; and, in less than six weeks after, she was running about the wards rosy and well, and has continued so up to the present time—about two months more.—*London Lancet.*

